# Epidermal Barrier Parameters in Psoriasis: Implications in Assessing Disease Severity

**DOI:** 10.3390/jpm14070728

**Published:** 2024-07-05

**Authors:** Silviu-Horia Morariu, Ovidiu Simion Cotoi, Oana Mirela Tiucă, Maria Crișan, Liuba Garaga, Robert Aurelian Tiucă, Claudia Raluca Mariean, Florin Corneliu Buicu, Alin Codrut Nicolescu

**Affiliations:** 1 Dermatology Department, George Emil Palade University of Medicine, Pharmacy, Science, and Technology of Targu Mures, 540142 Targu Mures, Romania; 2 Pathophysiology Department, George Emil Palade University of Medicine, Pharmacy, Science, and Technology of Targu Mures, 540142 Targu Mures, Romania; 3 Iuliu Haţieganu University of Medicine and Pharmacy, 400012 Cluj-Napoca, Romania; 4 Dermatology Clinic, Mures Clinical County Hospital, 540342 Targu Mures, Romania; 5 Endocrinology Department, George Emil Palade University of Medicine, Pharmacy, Science, and Technology of Targu Mures, 540142 Targu Mures, Romania; 6 Department of Public Health, George Emil Palade University of Medicine, Pharmacy, Science, and Technology of Targu Mures, 540142 Targu Mures, Romania; 7 Agrippa Ionescu Emergency Clinical Hospital, 011773 Bucharest, Romania

**Keywords:** psoriasis, skin barrier, hydration, tewl, emollients

## Abstract

Psoriasis is characterized by an aberrant immune response due to myeloid dendritic cells and T helper cells intertwining with keratinocyte hyperproliferation. Skin integrity alterations may predispose patients to physiological imbalances, such as xerosis, reduced elasticity, and increased friability. This study aims to assess the epidermal barrier dysfunction in chronic plaque psoriasis and gain a comprehensive view of the dynamic changes in the epidermal barrier during various topical therapies. Adult patients with chronic plaque psoriasis were enrolled in this observational study. For each patient, skin barrier parameters, stratum corneum hydration (SCH), transepidermal water loss (TEWL), elasticity, erythema, and melanin levels were measured in lesional and non-lesional skin. Two extensions of the initial study design, with subsequent epidermal barrier determinations, were made as follows: one in which patients with moderate psoriasis were treated with clobetasol propionate 0.5% and the second one in which mild psoriasis was treated with either clobetasol propionate 0.5% or clobetasol propionate 0.5% with 10% urea. TEWL and erythema were found to be higher in the sites affected by psoriatic lesions than the unaffected sites, while SCH and elasticity were decreased. Severe psoriasis presented with higher TEWL (*p* = 0.032), erythema (*p* = 0.002), and lower SCH (*p* < 0.001) compared with the mild and moderate forms. SCH significantly improved during clobetasol propionate 0.5% treatment (*p* = 0.015). Clobetasol propionate 0.5% with 10% urea was found to be superior to clobetasol propionate 0.5% in improving TEWL and SCH in psoriasis.

## 1. Introduction

Psoriasis is an immune-mediated disorder frequently associated with impaired life quality and various comorbidities, such as cardiovascular events, metabolic syndrome, and psychiatric disorders [1]. With complex etiopathogenesis, psoriasis lesions seem to be due to concomitant genetic [2,3], environmental [4], and aberrant immune responses [5].

Various treatment options are available nowadays for treating psoriasis, ranging from topical to classical systemic immunosuppressors and biologics [6], and they are selected mainly based on disease extension and severity. However, it should be noted that patients with psoriasis should benefit from a multidisciplinary approach and a tailored choice of treatment, considering the increased risk of comorbidities associated with this disease. However, it is not yet fully understood which type of patient would benefit from a specific type of treatment, highlighting once more the need for a comprehensive assessment of such patients and a closely monitored follow-up.

Psoriasis is characterized by an aberrant immune response due to myeloid dendritic cells and T helper cells intertwined with keratinocytes hyperproliferation, which further produce cytokines and chemokines and recruit immune cells, thereby sustaining and promoting an increased inflammatory state [7]. The immune system dysregulation, leading to an inflammatory microenvironment, further engages a heterogeneity in the immune cell population and various pathogenic activating signaling pathways [8], leading to multiple clinical phenotypes [9]. Various cytokines, such as interleukin (IL)-17, IL-12, IL-23, and tumor necrosis factor-α (TNF-α), are considered hallmarks of psoriasis etiopathogenesis [10]. Moreover, additional cytokines promoting psoriasis lesions, such as IL-20 and IL-8 [11] have been described, while IL-10 seems to exert antipsoriatic activity by inhibiting macrophage activity and the production of dendritic cells [12]. Currently, various agents, such as biologics or small-molecule inhibitors, targeting specific key pathogenetic interleukins are available for treating moderate-to-severe psoriasis [6], offering a comprehensive multimodal approach to the full spectrum of psoriatic disease, improving cutaneous lesions and alleviating associated comorbidities as well. Anti-IL-17 (ixekizumab, secukinumab, and brodalumab) and anti-IL-23 agents (risankizumab and guselkumab) are significantly linked to reaching a psoriasis area severity index (PASI) of 90 compared to the anti-IL12/23 agent, ustekinumab, while among the anti-TNF-α agents, infliximab surpasses adalimumab, etanercept, certolizumab in reaching PASI90 [6]. However, treatment choice should take into account, apart from disease severity and extension, the patient’s history, comorbidities, and perspective.

Keratinocyte hyperproliferation leads to an increased thickness of the epidermis, a decrease in the expression of proteins of the tight junction [13], as well as altered ceramide production in the extracellular matrix [14]. Therefore, changes in the epidermal barrier parameters are due to keratinocyte hyperproliferation. As seen in psoriasis, skin integrity alterations may predispose patients to physiological imbalances, such as xerosis, reduced elasticity, and increased friability.

Hydration of the stratum corneum (SCH) is one of the most important parameters in assessing the skin barrier. By definition, it comprehensively refers to the water content of the skin. Low SCH has been proven to be associated with a number of skin disorders, such as eczema or atopic dermatitis [15]. Transepidermal water loss (TEWL), another important parameter of the skin barrier, and defined by the flow density of water diffusing from deeper skin levels, such as the dermis and lower epidermis, to the skin surface, is also impaired in various cutaneous disorders. An elevated TEWL is associated with chronic skin diseases but is also impacted by age, sex, and sun exposure [16].

This study aims to assess the epidermal barrier dysfunction in chronic plaque psoriasis and gain a comprehensive view of the dynamic changes in the epidermal barrier during various topical therapies. To the best of our knowledge, this is the first study conducted on epidermal barrier parameters in Romanian patients suffering from psoriasis and the first to rely on comparator active treatment to assess dynamic epidermal barrier parameters.

## 2. Materials and Methods

### 2.1. Study Population

Patients suffering from chronic plaque psoriasis and admitted to the Dermatology Clinic of Mures Clinical County Hospital between May 2022 and January 2024 were included in this observational study. Data on the demographics, disease extension, severity and onset, and associated comorbidities were collected for each subject. Patients undergoing any topical and systemic therapy at the time of the analysis or two weeks prior were excluded from the study. Similarly, pediatric patients, those with incomplete data, who did not consent to the inclusion in the study, or those suffering from other dermatological disorders associated with xerosis were not included.

### 2.2. Study Design

After signing the informed consent, the patients’ cutaneous parameters were evaluated using the DermaLab Combo (Cortex Technology, Aalborg, Denmark) skin analysis system. The following parameters were determined on both lesional and non-lesional skin using designated probes for all included patients: stratum corneum hydration (SCH) (expressed in arbitrary units), TEWL (expressed in g/m^2^/h), elasticity (MPa for elasticity), and skin color measurement (erythema and melanin, expressed in arbitrary units). For all patients, the analysis was performed on non-sun-exposed skin to limit the possible cumulative damage caused by sun exposure on skin characteristics, such as hydration or elasticity. As such, determinations were performed on lesional and non-lesional skin from the lower back and, if unavailable, from the patient’s abdomen or upper thigh. Elasticity was determined twice, consecutively, on areas located five centimeters apart. At the same time, the other variables were measured five times each; all were reported in the analysis as the average of obtained results. All patients were examined in the same room, where ambient temperature was measured and maintained between 21 and 23 °C.

Next, additional determinations were performed to assess the cutaneous parameters’ changes during treatment for the patients with mild and moderate psoriasis who agreed to participate in an extension of the initial analysis. Patients eligible for topical treatment were divided into two study groups in this phase. The first group included patients with moderate psoriasis who were recommended to treat all their lesions with clobetasol propionate 0.5 mg/g ointment once a day. The second group included patients with mild psoriasis presenting with quasisymmetric lesions on non-sun-exposed skin and who agreed to treat the left part of their body with clobetasol propionate 0.5 mg/g ointment once a day and the right part of their body with clobetasol propionate 0.5 mg/g ointment with 10% urea. Afterward, both groups were clinically, and by means of imaging, followed up as follows: at baseline, at one week, two weeks, and one month. For each patient, the same cutaneous parameters were recorded (TEWL, SCH, elasticity, and skin color) at each visit following the aforementioned work protocol, and the PASI score was calculated.

**Figure 1:**
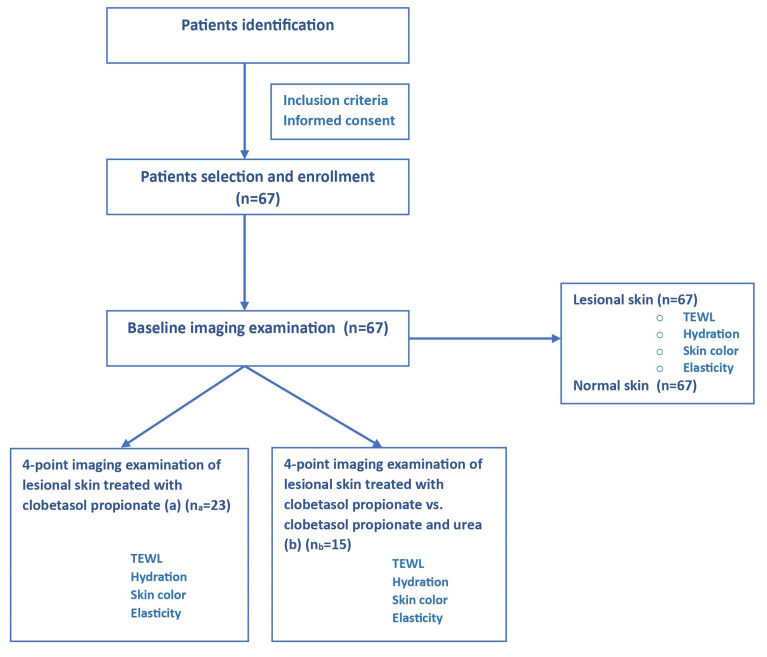
Study design.

The same investigator assessed disease severity for the same patient’s follow-up, and the PASI score was calculated. Disease severity was defined as follows: mild (PASI > 5), moderate (5 ≤ PASI ≤ 10), and severe (PASI ≥ 10). A schematical representation of the study design is depicted in Figure 1.

### 2.3. Statistical Analysis

The statistical analysis was performed with the MedCalc statistic software, version 22.023. Qualitative data were expressed as absolute values and percentages, while quantitative data were expressed as mean ± standard deviation (SD) or median values with 95% confidence intervals. Normality was assessed using the Shapiro–Wilk test, and subsequently, either the Wilcoxon test or t-test for paired samples was applied accordingly. Correlation between various parameters was analyzed using Spearman’s rank correlation. The Kruskal–Wallis test for non-normally distributed data or ANOVA one-way test for normal data, with subsequent post hoc Dunn–Bonferroni corrections, was performed in order to evaluate differences between study subgroups. A *p*-value of less than 0.05 was considered to be significant.

### 2.4. Study Outcome

The primary endpoint of our study was to assess differences in the cutaneous parameters in lesional and non-lesional skin in psoriasis. Secondarily, we aimed to evaluate changes in skin parameters in the psoriatic plaque during the course of various topical therapies.

## 3. Results

A total of 67 patients fulfilling the inclusion criteria were enrolled in this study. Most of them were males (*n* = 37), and the mean age at enrollment was 48.43 ± 16.87 years. Most patients had severe psoriasis (*n* = 29), followed by moderate forms (*n* = 23), with a mean PASI score of 11.62 ± 3.7. Patients’ clinical characteristics are depicted in Table 1.

### 3.1. Comparison of Skin Parameters between Lesional and Non-Lesional Skin in Chronic Plaque Psoriasis

The cutaneous parameters between lesional and non-lesional skin in psoriasis at baseline were compared, as seen in Table 2. Psoriatic plaques are defined by significantly higher TEWL and erythema (*p* < 0.001), while SCH, elasticity, and melanin values were notably lower compared to normal, non-lesional skin (*p* < 0.0001).

Further analysis was conducted on the correlation between the cutaneous parameters and disease severity. As the disease progresses, erythema increases (rho = 0.255, *p* = 0.03), TEWL increases (rho = 0.189, *p* = 0.04), and hydration decreases (rho = −0.342, *p* = 0.004). No other statistically significant correlations were noted.

Next, a subsequent analysis was performed to identify possible differences in the aforementioned parameters in patients suffering from mild, moderate, and severe psoriasis. Significant differences across the three subgroups were noted regarding SCH, TEWL, and erythema, as depicted in Table 3.

**Figure 2:**
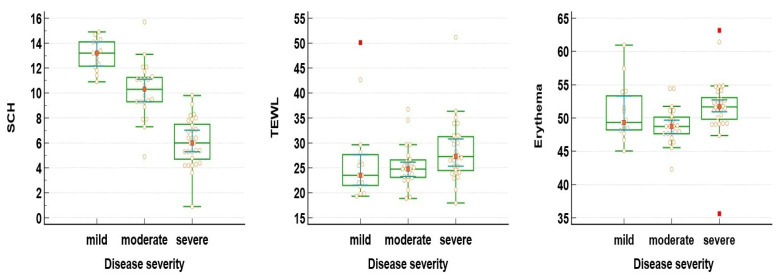
Pairwise comparison of SCH, TEWL, and erythema based on psoriasis severity.

Further, using post hoc Dunn–Bonferroni corrections, we noted that patients with severe psoriasis presented with significantly lower values for SCH than the mild and moderate forms. In terms of erythema, significantly higher values were noted in patients with severe psoriasis compared to moderate and mild forms (*p* < 0.05), but with no significant differences between the mild and moderate forms. Similarly, TEWL was significantly higher in severe psoriasis than in the moderate and mild forms (*p* = 0.032), as illustrated in Figure 2.

### 3.2. Lesional Skin Parameters Changes in Patients Undergoing Only Clobetasol Propionate 0.5% Ointment Treatment

Patients with moderate psoriasis were started on topical clobetasol propionate 0.5% ointment once a day on all the areas affected by psoriasis and monitored at the baseline, one-week, two-week, and one-month visits. Cutaneous parameters at the aforementioned visits are depicted in Table 4. TEWL and erythema significantly decreased during treatment with clobetasol propionate 0.5% ointment (*p* < 0.05), while SCH notably improved (*p* < 0.01). Moreover, subsequent correlations identified a significant and positive relationship between the erythema degree (rho = 0.22; *p*- = 0.03) and the PASI score, respectively, and a negative significant correlation between disease severity and SCH (rho = −0.52; *p* < 0.001).

**Figure 3:**
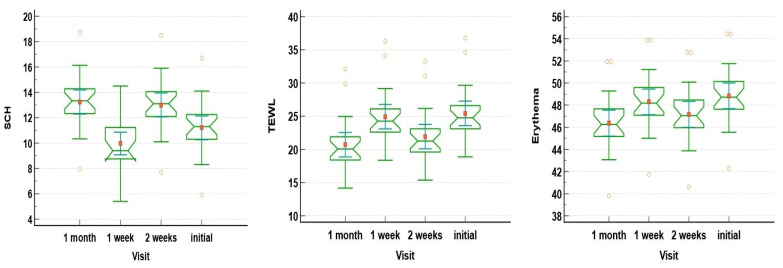
Pairwise comparison of SCH, TEWL, and erythema based on follow-up visits.

Next, Dunn–Bonferroni corrected post hoc analysis (Figure 3) highlighted that the SCH values varied significantly from visit to visit (*p* < 0.001), while TEWL decreased significantly from baseline towards the two-week hallmark (*p* < 0.015), but did not vary statistically significant (*p* = 0.06) thereafter. Erythema did not statistically significantly vary between the two-week visit or one-month visit and the baseline, respectively (*p* = 0.065 and 0.07); however, the erythema degree did notably improve since the initial visit when compared to the one-week and one-month follow-up.

### 3.3. Lesional Skin Parameters Variation in Patients Undergoing Clobetasol Propionate 0.5% Ointment Treatment vs. Patients Undergoing Clobetasol Propionate 0.5% Ointment with 10% Urea Treatment

Patients with mild psoriasis were started on a comparator topical treatment, as defined in the Methods section. Table 5 highlights comparative cutaneous parameter values at the four visits.

Patients in both study subgroups exhibited a significant decrease in TEWL and erythema, while SCH and melanin values increased during treatment. However, only SCH varied significantly between study groups when comparing epidermal barrier parameters at the one-month visit (*p* = 0.027). At the one-week follow-up, SCH varied significantly between the two study groups, while TEWL was significantly lower in the group treated with clobetasol propionate 0.5% and 10% urea at the two-week hallmark (Table 6).

## 4. Discussion

In our study, psoriatic plaque was defined by higher TEWL and erythema, while SCH, elasticity, and melanin levels were significantly lower compared to non-lesional skin. These results are in accordance with those published by Maroto-Morales et al. [17]. In most studies published so far, TEWL has been proven to be higher in psoriatic plaques than in non-lesional skin [18,19]. The imaging evaluation was performed on cloth-covered areas, such as the abdomen or the upper thigh, to limit the sun exposure effect on epidermal barrier parameters. Moreover, as measured by the PASI score, disease severity correlated significantly and positively with TEWL and erythema, indicating that skin permeability increases as the disease progresses, leading to increased water loss.

On the other hand, our study identified significantly lower SCH, elasticity, and melanin levels in the psoriatic plaque compared to normal-looking skin. Lower SCH values have been reported at areas with psoriatic plaques [20,21] than at uninvolved psoriatic skin areas and healthy controls, in agreement with our results. Previously, it was proven that the differences in TEWL and SCH values between lesional and non-lesional skin in psoriasis are due to decreased levels of aquaporin-3 (AQP3) expression [22]. Moreover, it seems that cutaneous AQP3 is increased by retinoic acid [23], a substance routinely used for treating psoriasis. It is thus safe to assume that by increasing AQP3 expression in psoriatic lesions, they will slowly subside. This is another powerful argument for the role that barrier dysfunction has in the onset and maintenance of psoriatic lesions.

Aberrant skin barrier parameters are important elements to consider when guiding patients’ therapy. Skin dryness is frequently linked to an impaired skin barrier. Patients should benefit from adjuvant moisturizers or emollients. While emollients restore the epidermal function by providing water and lipids and decrease cutaneous inflammation, moisturizers reduce skin water loss and hydrate the horny layer due to the fact that they contain humectants [24]. Psoriasis is associated with alterations in ceramides in the SC [25,26] and severely decreased natural moisturizing factors (NMF) [20].

Based on psoriasis severity, significant differences regarding SCH, TEWL, and erythema were also noted, indicating that skin changes in psoriasis are due to a continuous, dynamic process.

For the patients with moderate psoriasis who were started on clobetasol propionate 0.5% once daily for a month, a significant improvement in SCH, TEWL, erythema, and PASI score was noted. Moreover, these parameters correlated with the PASI score, indicating that they are useful parameters in assessing disease severity. TEWL varied significantly from baseline values to the two-week evaluation; afterward, even though a reduction was noted, it was not significant. The assessment of cutaneous parameters proves beneficial not only to assess baseline characteristics of patients’ lesions but also to provide important and objective information regarding one’s evolution under a specific choice of treatment.

Nowadays, potent and superpotent steroids are the mainstay of treatment for mild psoriasis and aid therapy in moderate-to-mild psoriasis. Lower-potency steroids are recommended for treating sensitive skin, such as the face or the intertriginous folds. Their action is due to both genomic, using the glucocorticoid receptor, and nongenomic pathways, with the latter being responsible for the fast action that the steroids possess [27]. Urea, an endogenous molecule, has multiple applications in dermatology. As a key element of the NMFs, it is an essential molecule for skin integrity and hydration. Even though it is a generally well-tolerated choice of therapy for most patients, caution should be taken when selecting the proper concentration. This is due to the fact that in low concentrations (up to 10%), it mainly acts as a humectant and increases skin hydration; in medium concentrations (up to 30%), it is both moisturizing and keratolytic; and at higher concentrations, it acts mainly as a keratolytic or debriding agent [28].

Patients with mild psoriasis who were started on the comparator treatment, clobetasol propionate 0.5% versus clobetasol propionate 0.5% and 10% urea, exhibited a significant decrease in all parameters, apart from elasticity, in both groups. When comparing these two treatment options, it was noted that SCH improvement was significantly higher in the group undergoing treatment with clobetasol propionate 0.5% and 10% urea at both the two-week follow-up and the one-month visit. TEWL improved significantly more in patients who underwent treatment with clobetasol propionate 0.5% and 10% urea compared to those solely on clobetasol propionate 0.5% at the two-week visit. Still, this difference was not noted at the one-month visit. In agreement with these data, we observed a decrease in TEWL values after adding 10% urea to the clobetasol propionate 0.5% ointment, which may be explained by its immediate barrier-repairing effect in a lipid-depleted epidermis. Likewise, Draelos et al. [29] observed a significant increase in skin hydration in 30 patients with psoriasis treated for 4 weeks using a moisturizing cream. Adding a humectant, such as 10% urea, to a topical active formulation may cause higher increases in SCH and therefore prove beneficial. Adding urea to patients’ treatment plans seems to aid in the remission of cutaneous lesions by decreasing epidermal proliferation [30], lowering the DNA-synthesis index, and reducing the number of keratinocytes in the cell cycle [31].

In our study, erythema slightly increased in both comparator treatment groups from the one-week visit to the two-week visit, most likely due to a physical effect. After the emollient was applied, the superficial scales were removed, and the deeper erythema was exposed. Nevertheless, even though, after the two-week examination, a linear decrease in erythema values was noted, the difference between the two study subgroups was not significant (*p* = 0.09), indicating that by adding urea, no marked improvement was noted in the erythema values. However, SCH and TEWL varied significantly between the two study subgroups, as depicted in Table 6 indicating that these are the two parameters mostly improved by adding urea to patients’ active treatment. Dynamic monitoring of epidermal barrier parameters should prove beneficial in tailoring therapeutic decisions and patient follow-up. Furthermore, even though high-potency steroids play a significant role in psoriasis treatment, they are frequently linked to skin atrophy, ulcerations [32], and withdrawal syndrome manifested by edema and erythema flares [33]. Epidermal barrier parameters may serve as useful indicators of disease evolution and should be taken into consideration to assist clinicians in minimizing steroids’ prolonged, unnecessary use.

This study was subject to several limitations. Skin homeostasis parameters are dependent on external factors; in order to limit this, the ambient conditions were also measured in order to increase the reliability of the results and all participants were examined in the same ambient conditions. Regarding the humectant and keratolytic agents, only the additional urea’s effect was tested. Future prospective studies enrolling more patients should prove beneficial since sample size also impacts the statistical parameters to a certain extent. Further research might focus on the impact of other substances on the skin barrier function of psoriasis patients in order to evaluate their effectiveness in improving the skin barrier function, not only in psoriasis but also in other chronic skin conditions. The skin barrier parameters were tested only with respect to chronic plaque psoriasis; future ideas might refer to evaluation of these parameters in regard to other psoriasis subtypes since there is a difference in the lamellar bodies and extracellular lamellar material [34] from clinical form to clinical form, which might further impact TEWL.

## 5. Conclusions

Among the psoriatic patients, TEWL and erythema were found to be higher in sites affected by psoriatic lesions than the unaffected sites, while SCH and elasticity were decreased. As TEWL and SCH measure skin hydration and barrier function, they may also indicate an impaired skin barrier function and reduced skin hydration. Clobetasol propionate 0.5% with 10% urea is superior to clobetasol propionate 0.5% in improving TEWL and SCH in psoriasis. We can safely argue that, apart from being characterized by immunological alterations, psoriasis is also defined by a defective skin barrier thus requiring comprehensive skin hydration for better management of skin lesions akin to atopic skin.

## Figures and Tables

**Table 1:** Patients’ clinical characteristics.

Parameter	All Patients (*n* = 67)	Mild Psoriasis (*n* = 15)	Moderate Psoriasis (*n* = 23)	Severe Psoriasis (*n* = 29)
Gender				
Male	37	12	14	11
Female	30	3	9	18
Employment status				
Employed	45	15	14	16
Unemployed/retired	22	0	9	13
BMI values (kg/m^2^)	27.46 ± 7.32	24.56 ± 4.34	25.6 ± 7.34	30.6 ± 6.85
Comorbidities				
Hypertension	35	6	12	17
Diabetes	18	2	6	12
Dyslipidemia	27	3	6	18
Joint involvement (psoriatic arthritis)	21	1	6	14
Eye involvement (uveitis)	3	0	0	3
Special sites				
Scalp involvement	32	2	12	18
Nail involvement	30	3	11	16
Genital involvement	3	0	1	2

BMI, body mass index.

**Table 2:** Skin barrier parameters in normal skin versus psoriatic plaque.

Parameter	Normal Skin	Psoriatic Plaque	*p*-Value
SCH	33.4 [28.90–37.38]	9.3 [7.9–10.59]	<0.0001
TEWL	10.3 [9.70–10.79]	25.71 [24.76–27.03]	<0.0001
Elasticity	7.71 [7.36–8.27]	6.7 [6.40–7.19]	<0.0001
Erythema	28.16 [27.78–28.44]	50.30 [49.12–51.26]	<0.0001
Melanin	26 [25.73–27.34]	19.4 [18.90–20.09]	<0.0001

SCH, stratum corneum hydration; TEWL, transepidermal water loss.

**Table 3:** Epidermal barrier parameters based on disease severity.

Parameter	Mild Psoriasis (*n* = 15)	Moderate Psoriasis (*n* = 23)	Severe Psoriasis (*n* = 29)	*p*-Value
SCH	12.10 [11.10–13.12]	7.90 [6.60–9.23]	5.3 [4.25–6]	<0.001
TEWL	23.5 [21.49–27.64]	24.76 [23.29–26.14]	27.29 [25.32–30.77]	0.032
Elasticity	7.2 [6.13–7.4]	6.4 [5.70–6.76]	7.1 [6.58–7.52]	0.370
Erythema	48.72 [47.66–49.68]	49.34 [48.21–53.39]	51.67 [50.96–52.71]	0.002
Melanin	18.4 [17.40–19.77]	19.4 [18.9–20.66]	19.4 [18.78–20.91]	0.217

SCH, stratum corneum hydration; TEWL, transepidermal water loss; per the Kruskal–Wallis test.

**Table 4:** Variations in the epidermal barrier parameters during treatment with clobetasol propionate.

Parameter	Initial Visit	1-Week Visit	2-Weeks Visit	1-Month Visit	*p*-Value
SCH	11.21 ± 2.18	9.96 ± 2.06	13.01 ± 2.18	13.23 ± 2.18	<0.01
TEWL	25.41 ± 4.24	24.91 ± 4.24	21.91 ± 4.24	20.71 ± 4.24	<0.001
Elasticity	6.60 ± 1.15	6.95 ± 1.15	6.80 ± 1.15	7.25 ± 1.15	0.284
Erythema	48.83 ± 2.69	48.29 ± 2.69	47.16 ± 2.69	46.36 ± 2.69	0.011
Melanin	19.87 ± 1.84	20.12 ± 1.84	20.16 ± 1.84	20.41 ± 1.84	0.804
PASI	7.53 ± 1.61	6.78 ± 1.42	6.17 ± 1.62	3.73 ± 1.65	<0.001

SCH, stratum corneum hydration; TEWL, transepidermal water loss; PASI, psoriasis area severity index; per ANOVA one-way test.

**Table 5:** Epidermal barrier parameter variations in patients undergoing comparator treatment.

Parameter	Initial Visit	1-Week Visit	2-Weeks Visit	1-Month Visit	*p*-Value
Clobetasol propionate 0.5%					
SCH	11.2 [10.15–12.10]	9.2 [8.55–10.04]	12.6 [12.10–13.90]	14.13 [12.03–14.27]	<0.001
TEWL	23.5 [21.49–27.64]	24.25 [22.05–25.66]	22.01 [18.97–26.74]	20.05 [18.40–22.56]	0.014
Elasticity	7.2 [6.12–7.40]	6.65 [5.95–7.12]	6.9 [6.01–7.69]	7.35 [6.62–8.01]	0.37
Erythema	49.34 [48.21–53.29]	47.37 [46.60–48.92]	47.53 [46.45–49.23]	46.25 [45.18–47.08]	<0.001
Melanin	18.4 [17.40–19.76]	20.05 [19.25–21.49]	19.44 [18.85–20.75]	22.32 [19.98–23.59]	0.015
Clobetasol propionate 0.5% and 10% urea					
SCH	10.95 [9.90–11.75]	11.55 [10.50–12.45]	12.43 [11.97–14.04]	14.73 [12.03–18.40]	<0.001
TEWL	23.35 [21.34–27.49]	22.6 [20.59–26.74]	21.25 [19.24–22.66]	18.58 [16.97–22.44]	0.027
Elasticity	7.4 [6.32–7.60]	7.35 [6.26–7.55]	6.9 [5.80–7.43]	7.35 [6.56–8.21]	0.54
Erythema	49.94 [48.81–53.89]	48.44 [47.21–50.64]	48.98 [46.28–49.91]	47.21 [45.46–49.24]	0.045
Melanin	17.62 [16.62–18.98]	18.4 [17.40–19.76]	20.03 [18.40–21.35]	20.94 [19.98–21.59]	0.002

SCH, stratum corneum hydration; TEWL, transepidermal water loss. per the Kruskal–Wallis test.

**Table 6:** Comparison of epidermal barrier parameters between the groups on comparator treatment.

Parameter	Clobetasol Propionate 0.5% vs. Clobetasol Propionate 0.5% and 10% Urea		
	1 Week	2 Weeks	1 Month
	*p*-Value	*p*-Value	*p*-Value
SCH	0.003	0.62	0.027
TEWL	0.804	0.043	0.07
Elasticity	0.569	0.224	0.499
Erythema	0.254	0.138	0.09
Melanin	0.07	0.09	0.46

SCH, stratum corneum hydration; TEWL, transepidermal water loss.

## Data Availability

All data presented can be made available upon reasonable request.
